# P05 Comparing patients’ and healthcare professionals’ experiences and views on the management of recurrent urinary tract infections: qualitative evidence synthesis and meta-ethnography

**DOI:** 10.1093/jacamr/dlad077.009

**Published:** 2023-08-02

**Authors:** Leigh Sanyaolu, Catherine Hayes, Donna Lecky, Alison Weightman, Harry Ahmed, Rebecca Cannings-John, Adrian Edwards, Fiona Wood

**Affiliations:** Cardiff University, Cardiff, UK; UK Health Security Agency, UK; Cardiff University, Cardiff, UK; UK Health Security Agency, UK; Cardiff University, Cardiff, UK; Cardiff University, Cardiff, UK; Cardiff University, Cardiff, UK; Cardiff University, Cardiff, UK; Cardiff University, Cardiff, UK

## Abstract

**Background:**

Urinary tract infections (UTIs) are common and result in significant morbidity, negative impacts on daily life, reduced quality of life and reduced work attendance. Recurrence is common and recurrent UTIs have an estimated annual prevalence of 3%. The experience of women with recurrent UTIs is not well understood and how this compares to the views of healthcare professionals (HCPs) is unknown.

**Objectives:**

This qualitative evidence synthesis aims to understand the experiences of women with recurrent UTIs and compare them to primary care HCPs.

**Methods:**

We systematically searched MEDLINE, Embase, CINAHL, PsychInfo, ASSIA, Web of Science and the grey literature from inception to June 2022 for primary qualitative studies. Meta-ethnography was conducted to synthesize the studies and the findings for women with recurrent UTIs and HCPs were then compared.

**Results:**

Twelve primary qualitative studies published from 2005 to 2022 and conducted in Europe and the USA were included. Two studies involved primary care staff; nine studies involved women with experience of recurrent UTIs. One study involved both. Patients and HCPs had similar views in terms of the causes of recurrent UTIs and self-management and similar concerns about an underlying cause and antibiotic use. They also had similar views on the impact of recurrent UTIs, however patients felt HCPs did not appreciate the impact of recurrent UTIs. There were conflicts in terms of the expectation for antibiotics and the need for further investigation and referral.

**Conclusions:**

This is the first qualitative evidence synthesis on the experiences of women with recurrent UTIs and the views of primary care HCPs. It demonstrates that women with recurrent UTIs and HCPs share several similar views and concerns but there were significant communication gaps. Further guidance development and a patient decision aid could help address these gaps.

